# Cyclooxygenase (COX)-2 modulates *Toxoplasma gondii* infection, immune response and lipid droplets formation in human trophoblast cells and villous explants

**DOI:** 10.1038/s41598-021-92120-3

**Published:** 2021-06-16

**Authors:** Guilherme de Souza, Rafaela José Silva, Iliana Claudia Balga Milián, Alessandra Monteiro Rosini, Thádia Evelyn de Araújo, Samuel Cota Teixeira, Mário Cézar Oliveira, Priscila Silva Franco, Claudio Vieira da Silva, José Roberto Mineo, Neide Maria Silva, Eloisa Amália Vieira Ferro, Bellisa Freitas Barbosa

**Affiliations:** 1 Laboratory of Immunophysiology of Reproduction, Institute of Biomedical Science, Federal University of Uberlândia, Campus Umuarama, Av. Pará, 1720, Uberlândia, MG 38405-320 Brazil; 2 Laboratory of Immunopathology, Institute of Biomedical Sciences, Federal University of Uberlândia, Uberlândia, Brazil; 3 Laboratory of Trypanosomatids, Institute of Biomedical Sciences, Federal University of Uberlândia, Uberlândia, Brazil; 4 Laboratory of Immunoparasitology, Institute of Biomedical Sciences, Federal University of Uberlândia, Uberlândia, Brazil

**Keywords:** Infectious diseases, Immunology, Antimicrobial responses, Cytokines, Infection, Infectious diseases

## Abstract

Congenital toxoplasmosis is represented by the transplacental passage of *Toxoplasma gondii* from the mother to the fetus. Our studies demonstrated that *T. gondii* developed mechanisms to evade of the host immune response, such as cyclooxygenase (COX)-2 and prostaglandin E_2_ (PGE_2_) induction, and these mediators can be produced/stored in lipid droplets (LDs). The aim of this study was to evaluate the role of COX-2 and LDs during *T. gondii* infection in human trophoblast cells and villous explants. Our data demonstrated that COX-2 inhibitors decreased *T. gondii* replication in trophoblast cells and villous. In BeWo cells, the COX-2 inhibitors induced an increase of pro-inflammatory cytokines (IL-6 and MIF), and a decrease in anti-inflammatory cytokines (IL-4 and IL-10). In HTR-8/SVneo cells, the COX-2 inhibitors induced an increase of IL-6 and nitrite and decreased IL-4 and TGF-β1. In villous explants, the COX-2 inhibitors increased MIF and decreased TNF-α and IL-10. Furthermore, *T. gondii* induced an increase in LDs in BeWo and HTR-8/SVneo, but COX-2 inhibitors reduced LDs in both cells type. We highlighted that COX-2 is a key factor to *T. gondii* proliferation in human trophoblast cells, since its inhibition induced a pro-inflammatory response capable of controlling parasitism and leading to a decrease in the availability of LDs, which are essentials for parasite growth.

## Introduction

Toxoplasmosis is a disease caused by *Toxoplasma gondii*, an obligate intracellular protozoan parasite that can infect all warm-blooded vertebrates, such as birds and mammals, including humans, representing a significant public health problem^1,2^. It is estimated that 30 to 50% of the world population is infected with the parasite^3^. Congenital toxoplasmosis is considered one of the most severe forms of the disease, and occurs due to the transplacental passage of *T. gondii* tachyzoites during pregnancy, reaching circulation and fetal tissues, which may have serious implications from fetus to adulthood^4–6^.

The immune response against *T. gondii* is primarily mediated by T helper 1 (Th1) cells, and requires components of the innate and adaptive immune response^7^. At the beginning of the infection, *T. gondii* is recognized by innate imune response cells, stimulating synthesis of interleukin (IL)-12 in dendritic, macrophage and neutrophil cells, and induces interferon (IFN)-γ production by natural killer (NK) cells. Additionally, tumor necrosis factor (TNF) can act in synergism with IL-12, optimizing IFN-γ production by these cells^8,9^. The production of these pro-inflammatory cytokines is associated with activation of the CD4 and CD8 T-lymphocyte-mediated adaptive immune response, which act to produce and secrete various inflammatory mediators, such as nitric oxide (NO), and induce an even greater increase in the levels of IL-12 and IFN-γ^10,11^. IFN-γ activates dendritic cells, macrophages and neutrophils, promoting the reduction or elimination of *T. gondii*. When macrophages are activated by IFN-γ, there is an increase in NO production, promoting a toxic action against the parasite^12^. In addition to IFN-γ and IL-12, other pro-inflammatory cytokines are important to control *T. gondii* infection, such as IL-6 and macrophage migration inhibition fator (MIF)^13–15^. However, an exacerbated inflammatory response can lead the host to death^8,16^. Thus, it is important to have a balance between a Th1 and Th2 profile. This balance can be mediated by anti-inflammatory cytokines production, such as IL-4, IL-10 and transforming growth fator (TGF)-β1, which act by decreasing NO production in macrophages and cytotoxic activity of NK cells^17,18^. In pregnancy, this immune regulation mediated by anti-inflammatory and regulatory cytokines is important for the developmnet of the placenta and protection of the semiallogeneic fetus from maternal immune response^19,20^. However, this environment characterized by a preferential Th2 profile contributes to infection by *T. gondii*, which can cause congenital toxoplasmosis^21^.

Lipid droplets (LDs) are lipid-rich intracellular organelles present in all eukaryotic cells and bacteria^22^. LDs are involved in a variety of functions, such as lipid metabolism, cell signaling, and they are found in inflammatory and infectious conditions^23–25^. In recent years, several studies have shown that many pathogens target LDs, including viruses^26–30^, intracellular bacteria^31–34^ and protozoan^35–38^. These pathogens are known to seek lipid resources for their maintenance and survival, however studies have showed that LDs also can be central mediators of immune responses^22,39^. It was demonstrated that *T. gondii* induces LDs production in the host cell for own maintenance and survival^37,40,41^. Also, it was shown that the ability to increase LDs production was conserved in three *T. gondii* strains (I—RH, II—ME-49; III- CEP)^40^.

*T. gondii* is known to be auxotrophic for cholesterol, and studies show that the parasite can capture this low-density lipoprotein (LDL)-derived cholesterol from the host cell^42,43^. Once internalized by the parasite, this cholesterol can be esterified and stored in LDs for maintenance and replication of the parasite^43^. However, the capture of these lipid droplets represents a major challenge for pathogens, since the fatty acids stored inside are used by mitochondria to produce energy through β-oxidation^41^. In this sense, Pernas and collaborators^44^ showed that *T. gondii* infection in mouse embryonic fibroblast (MEF) cells triggered recruitment of mitochondria around the parasitophorous vacuole, which increased the uptake of fatty acids by these organelles and consequently decreases the uptake of lipids by *T. gondii*, suggesting that mitochondrial metabolism of the host cell during infection may control the growth of the parasite. Thus, *T. gondii* is a potential pathogen able to recruit and induce LDs formation in host cells for guarantee its own metabolism and survival^44,45^.

LDs are also important for the production of inflammatory mediators^46^. These organelles store arachidonic acid (AA), which is esterified into phospholipids and neutral lipids^46^. When the cell detects an appropriate stimulus, AA is released, a process mediated by cPLA_2_ (calcium dependent phospholipase A_2_), and converted into prostaglandin E_2_ (PGE_2_) by the enzyme called cyclooxygenase (COX)-2^47,48^. Studies show that LD biogenesis is related to a higher formation of COX-2 and PGE_2_^31,49,50^. In this sense, macrophages infected with *Trypanosoma cruzi* increased the formation of LDs, and it was associated with high levels of PGE_2_^51^. In other study, it was shown that high levels of PGE_2_ favored the growth of *T. cruzi* (Y strain) in BALB/c mice^52^. In contrast, COX-2 inhibitors by meloxicam or etoricoxib promoted a reduction of both the PGE_2_ release and *T. cruzi* amastigotes in the cardiac muscle, culminating in an increase of mice survival^52^. In addition, studies showed that murine peritoneal macrophages and skeletal muscle cells increased the amount of LDs when infected with *T. gondii*, which this formation correlates with increased PGE_2_ production and reduced NO release, indicating that COX-2 and PGE_2_ are potent Th1 response inhibitor^37,45^. Finally, studies from our group have shown that inhibition of COX-2 and PGE_2_ decreased *T. gondii* infection in *Calomys callosus* rodent, in peritoneal macrophages and in human monocytes^53^. Additionally, when PGE_2_ was added to human monocytes treated with COX-2 inhibitors, the parasite growth was restored, showing the importance of these inflammatory mediators for *T. gondii* proliferation^53^. Considering all these studies, it is possible to conclude that there is an association between LDs and the COX-2/PGE_2_ axis to favor infection by *T. gondii* and *T. cruzi* in host cells. However, there are no studies demonstrating the role of COX-2 in *T. gondii* proliferation and production of LDs in human trophoblast cells.

In this sense, this study aimed to evaluate the role of COX-2 in the susceptibility of *T. gondii* infection in villous and extravillous human trophoblast cells, as well as in human chorionic villous explants from the third trimester of pregnancy. Additionally, we also evaluated the functional role of COX-2 in the immune response and lipid droplets formation in these experimental models. Our research group has investigated the mechanisms of *T. gondii* infection in trophoblast cells using BeWo^54–57^ and HTR-8/SVneo^58–60^ cell lines as villous and extravillous trophoblast cells models, respectively, and human chorionic villous explants as maternal–fetal interface experimental model^56,60–62^.

## Results

### COX-2 inhibitors did not change the viability of BeWo and HTR-8/SVneo cells

**Figure 1:**
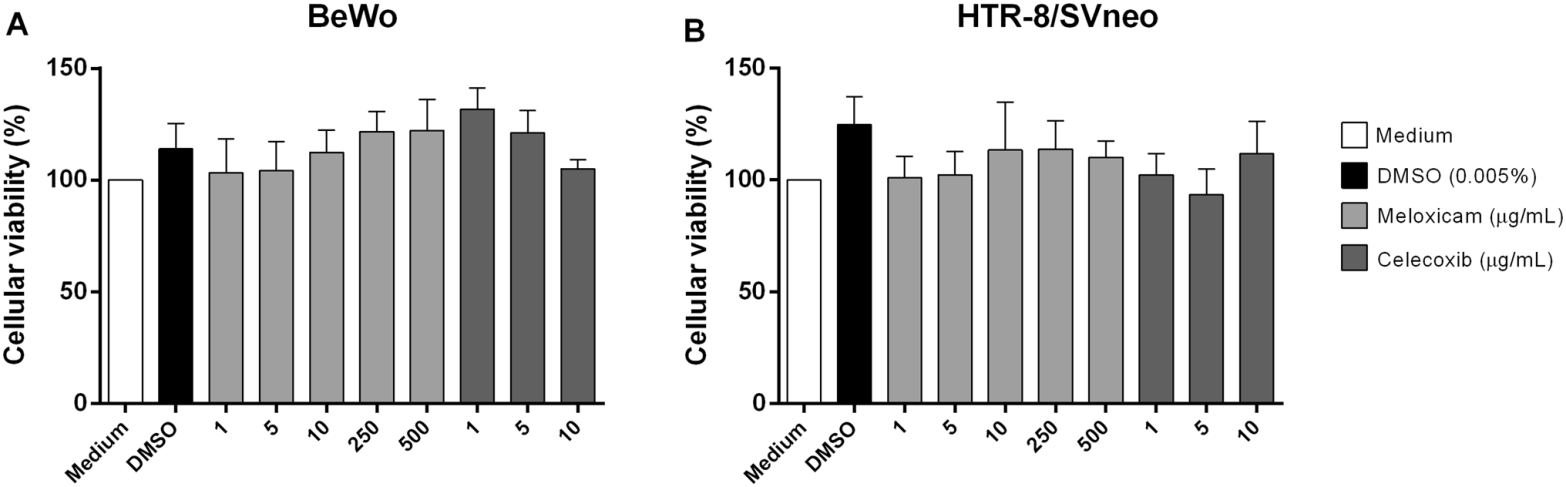
Cellular viability in BeWo and HTR-8/SVneo cells treated with COX-2 inhibitors. BeWo (**A**) and HTR-8/SVneo (**B**) cells were treated with meloxicam or celecoxib in increasing concentrations, or untreated (medium) or treated with 0.005% DMSO. After 24 h, the cells were submitted to MTT assay and data were presented as percentage (%) of viable cells (cellular viability) in relation to untreated cells (medium, 100% of cellular viability). Data were shown as mean ± SEM from three independents experiments with eight replicates. Differences between groups were analyzed by Kruskal–Wallis test and Dunn’s multiple comparison post-test (GraphPad Prism Sofware version 6.01, https://www.graphpad.com). Differences were considered significant when *P* < 0.05.

Initially, we evaluated the cellular viability of BeWo and HTR-8/SVneo cells treated with COX-2 inhibitors, meloxicam or celecoxib, at different concentrations (Fig. 1).

We observed that both inhibitors did not induce toxicity at any concentration tested when compared to untreated cells (medium), regardless of cell type. In addition, DMSO (0.005%) also did not trigger toxicity in both cells (Fig. 1A,B).

Based on these results, we chose two concentrations of each inhibitor for further experiments, one of low and one of high concentration. For meloxicam, 10 and 250 μg/mL were chosen, while for celecoxib 1 and 5 μg/mL were chosen.

### COX-2 inhibitors reduced *T. gondii* intracellular proliferation in BeWo and HTR-8/SVneo cells

**Figure 2:**
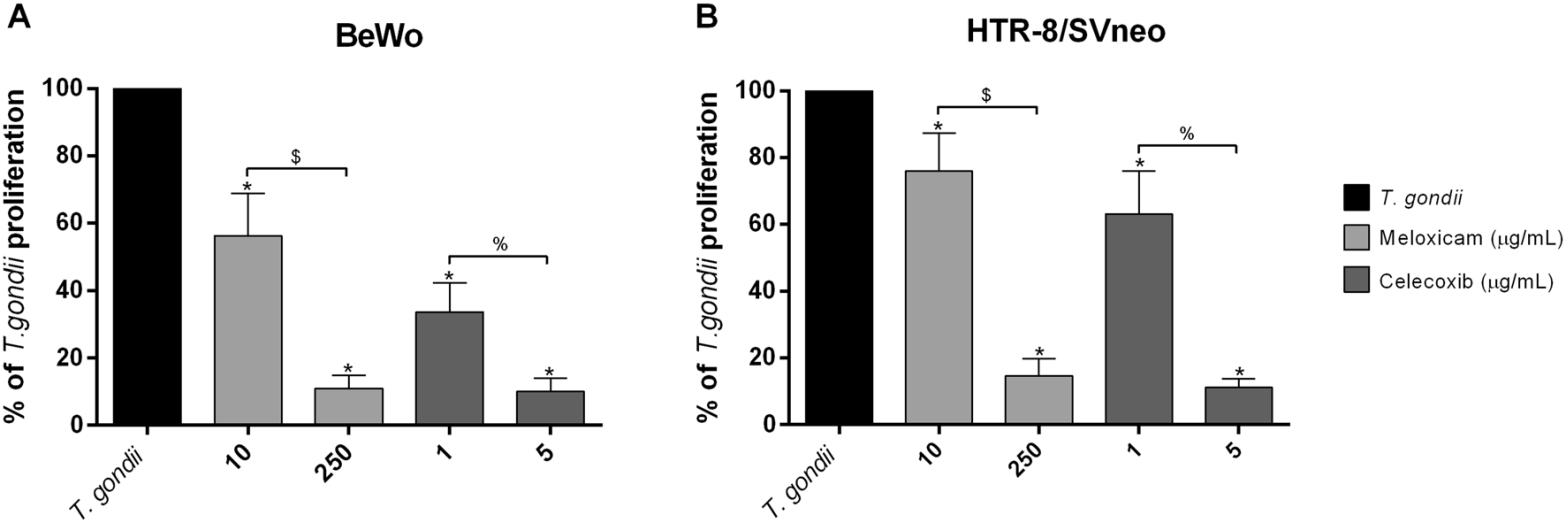
*T. gondii* intracellular proliferation in BeWo and HTR-8/SVneo cells treated with COX-2 inhibitors. BeWo (**A**) and HTR-8/SVneo (**B**) cells were infected with *T. gondii* for 3 h and treated or not with meloxicam or celecoxib for an additional 24 h. Next, cells were submitted to *T. gondii* intracellular proliferation assay (β-galactosidase assay), and data were presented as percentage (%) of *T. gondii* proliferation in relation to untreated and infected cells (100% of intracellular proliferation). Data were shown as mean ± SEM from three independents experiments with eight replicates. Differences between groups were analyzed by Kruskal–Wallis test and Dunn’s multiple comparison post-test (GraphPad Prism Sofware version 6.01, https://www.graphpad.com). Significant differences in relation to untreated and infected cells (^*^T. gondii), and between concentrations of meloxicam (^$^) or celecoxib (^%^). Differences were considered significant when *P* < 0.05.

Next, we evaluated the effect of COX-2 inhibitors on *T. gondii* intracellular proliferation in BeWo and HTR-8/SVneo cells (Fig. 2).

We observed that in BeWo cells, the meloxicam (10 μg/mL: ^*^P = 0.0448; 250 μg/mL: ^*^P < 0.0001) and celecoxib (1 μg/mL: ^*^P = 0.0056; 5 μg/mL: ^*^P < 0.0001) induced a reduction in *T. gondii* proliferation when compared to untreated and infected cells (*T. gondii*); moreover, for the half-maximum antiparasitic activity (IC_50_), meloxicam required 17.0 ± 9.2 μg/mL, while celecoxib demanded 0.3 ± 0.2 μg/mL. Furthermore, our results showed a significant difference between the differente doses of meloxicam (^$^*P* < 0.0001) or celecoxib (^%^*P* < 0.0094) (Fig. 2A).

In HTR-8/SVneo cells, the meloxicam (10 μg/mL: ^*^P = 0.0261; 250 μg/mL: ^*^P < 0.0001) and celecoxib (1 μg/mL: ^*^P = 0.0010; 5 μg/mL: ^*^P < 0.0001) also induced a reduction in *T. gondii* proliferation when compared to untreated and infected cells (*T. gondii*). In this cell line, meloxicam and celecoxib presented IC_50_ of 39.7 ± 13.2, and 1.5 ± 0.2 μg/mL, respectively. Furthermore, our results showed a significant difference between the different doses of meloxicam (^$^*P* = 0.0007) or celecoxib (^%^*P* = 0.0009) (Fig. 2B), showing that the inhibition was dose-dependent in both cells.

### COX-2 inhibitors increased IL-6 and MIF and decreased IL-4 and IL-10 in BeWo cells infected by *T. gondii*

**Figure 3:**
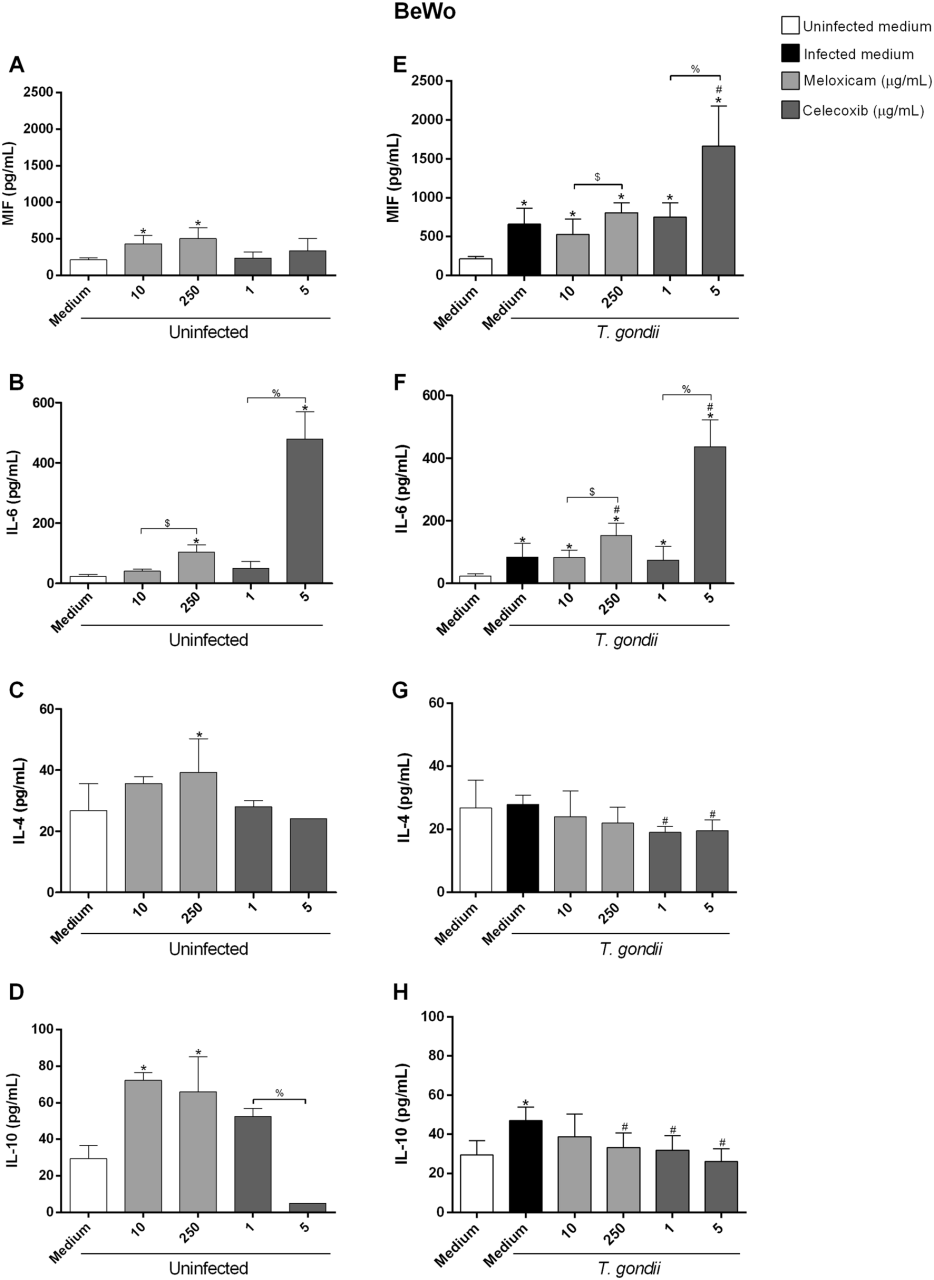
Cytokines production in BeWo cells infected and treated with COX-2 inhibitors. BeWo cells were infected or not with *T. gondii* for 3 h and treated or not with meloxicam or celecoxib for an additional 24 h. Next, the supernatants were collected for measurement of MIF (**A**, **E**), IL-6 (**B**, **F**), IL-4 (**C**, **G**) and IL-10 (**D**, **H**) by ELISA. Data were shown as mean ± SEM from three independents experiments with eight replicates. Differences between groups were analyzed by One-Way ANOVA test with Sidak’s multiple comparison (**A**, **B**, **E**, **F**) or Kruskal–Wallis test and Dunn’s multiple comparison post-test (**C**, **D**, **G**, **H**) (GraphPad Prism Sofware version 6.01, https://www.graphpad.com). Significant differences in relation to untreated and uninfected cells (^*^uninfected medium), untreated and infected cells (^#^infected medium), between concentrations of meloxicam (^$^) or celecoxib (^%^), regardless of infection. Differences were considered significant when *P* < 0.05.

Next, we evaluated the cytokine production in the supernatant of BeWo cells under different experimental situations (Fig. 3**)**.

Our data showed that uninfected cells and treated only with meloxicam (10 μg/mL: ^*^P = 0.004; 250 μg/mL: ^*^P < 0.0002) increased the MIF production when compared to untreated and uninfected cells (Fig. 3A). In addition, we observed an increase in MIF production in untreated and infected cells (^*^P = 0.0073) or infected and treated with meloxicam (10 μg/mL: ^*^P = 0.0164; 250 μg/mL: ^*^P = 0.0002) or celecoxib (1 μg/mL: ^*^P = 0.0015; 5 μg/mL: ^*^P < 0.0001) when compared to untreated and uninfected cells (Fig. 3E). In addition, the higher concentration of both inhibitors induced greater MIF production when compared to lower concentrations (meloxicam: ^$^*P* = 0.033; celecoxib: ^%^*P* < 0.0001) in treated and infected cells, showing a dose-dependent effect (Fig. 3E).

In relation to IL-6, we observed that uninfected cells and treated with the higher concentration of meloxicam (^*^P = 0.0005) or celecoxib (^*^P < 0.0001) increased the production of this cytokine when compared to untreated and uninfected cells, showing a dose-dependent effect (Fig. 3B). In untreated and infected cells (^*^P = 0.034) or infected and treated with meloxicam (10 μg/mL: ^*^P = 0.0431; 250 μg/mL: ^*^P < 0.0001) or celecoxib (1 μg/mL: ^*^P = 0.021; 5 μg/mL: ^*^P < 0.0001), there was an increased production of this cytokine when compared to untreated and uninfected cells (Fig. 3F). Also, we observed an increase in IL-6 production in infected cells and treated with higher concentration of meloxicam (^#^*P* = 0.0111) and celecoxib (^#^*P* < 0.0001) when compared to untreated and infected cells (Fig. 3F). Furthermore, the increase IL-6 production in treated and infected cells also had a dose-dependent effect (Fig. 3F).

In relation to IL-4, we observed that only uninfected cells and treated with meloxicam 250 μg/mL increased the production of this cytokine (^*^P = 0.0338) (Fig. 3C). In contrast, in infected cells and treated with both concentrations of celecoxib (1 μg/mL: ^#^*P* = 0.0195; 5 μg/mL: ^#^*P* = 0.0184), the IL-4 production decreased when compared to untreated and infected cells (Fig. 3G).

Regarding to IL-10, we observed that uninfected cells and treated with both concentrations of meloxicam (10 μg/mL: ^*^P = 0.0031; 250 μg/mL: ^*^P = 0.0107), there was an increased production of this cytokine when compared to untreated and uninfected cells, and a dose-dependent effect was observed in relation to celecoxib (Fig. 3D). In untreated and infected cells, we observed an increase in IL-10 production when compared to untreated and uninfected cells (^*^P = 0.0367) (Fig. 3H). However, infected cells and treated with 250 μg/mL of meloxicam (^#^*P* = 0.041) or both concentrations of celecoxib (1 μg/mL: ^#^*P* = 0.0303; 5 μg/mL: ^#^*P* = 0.0002) decreased the production of this cytokine when compared to untreated and infected cells (Fig. 3H).

In summary, our data show that COX-2 inhibitors increase MIF and IL-6, and decrease IL-4 and IL-10 production in BeWo cells, triggering a pro-inflammatory profile in BeWo cells.

IL-8, TGF-β1, TNF-α and nitrite showed values below detection levels in all conditions tested in BeWo cells (data not shown).

### COX-2 inhibitors increased IL-6 and nitrite and decreased IL-4 and TGF-β1 in HTR-8/SVneo cells infected by *T. gondii*

**Figure 4:**
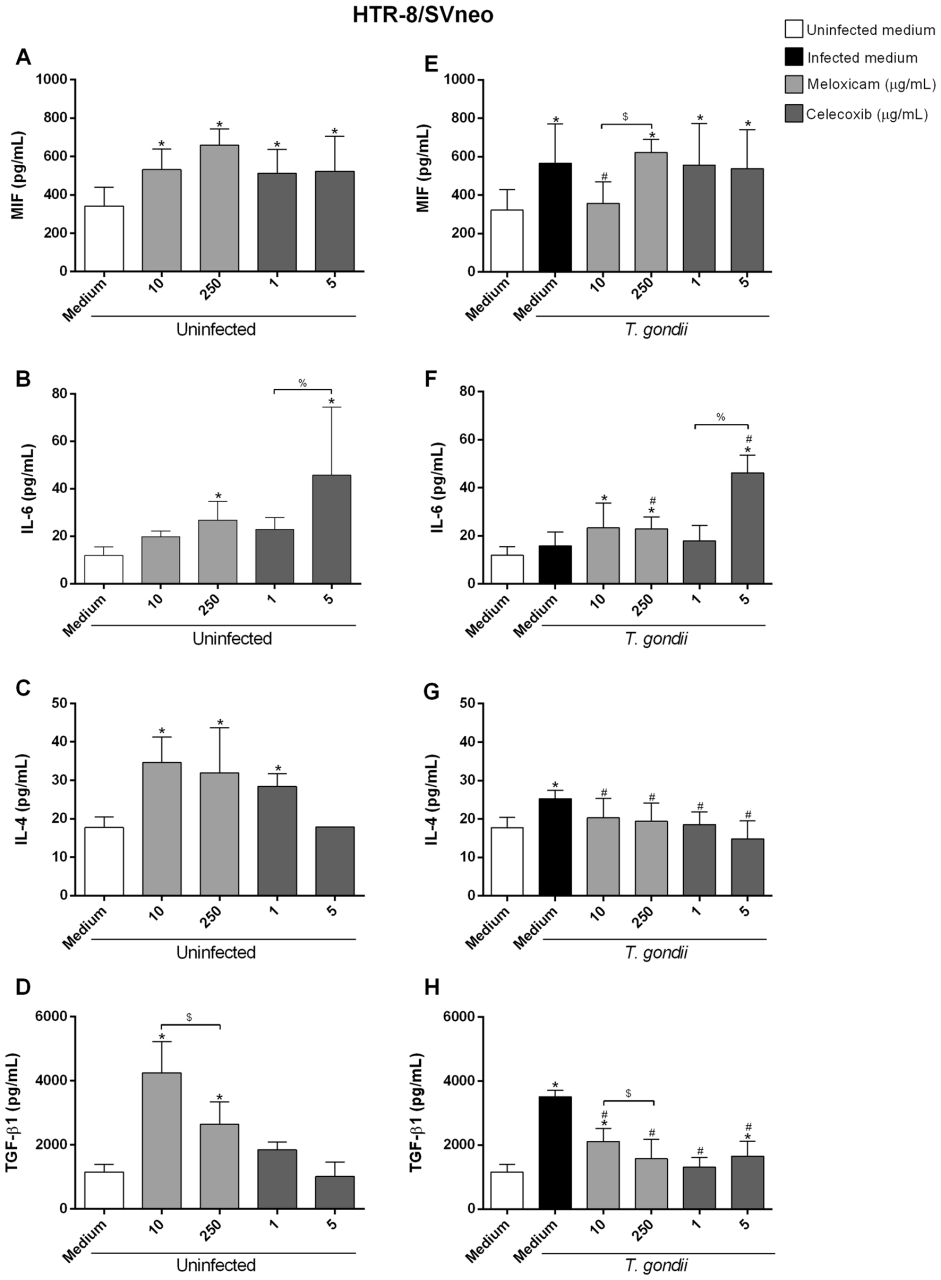
Cytokines production in HTR-8/SVneo cells infected and treated with COX-2 inhibitors. HTR-8/SVneo cells were infected or not with *T. gondii* for 3 h and treated or not with meloxicam or celecoxib for an additional 24 h. Next, the supernatants were collected for measurement of MIF (**A**, **E**), IL-6 (**B**, **F**), IL-4 (**C**, **G**) and TGF-β1 (**D**, **H**) by ELISA. Data were shown as mean ± SEM from three independents experiments with eight replicates. Differences between groups were analyzed by One-Way ANOVA test with Sidak’s multiple comparison (**A**, **B**, **D**, **F**, **G**, **H**) or Kruskal–Wallis test and Dunn’s multiple comparison post-test (**C**, **E**) (GraphPad Prism Sofware version 6.01, https://www.graphpad.com). Significant differences in relation to untreated and uninfected cells (^*^uninfected medium), untreated and infected cells (^#^infected medium), and between concentrations of meloxicam (^$^) or celecoxib (^%^), regardless of infection. Differences were considered significant when *P* < 0.05.

Next, we also evaluated the cytokine production and nitrite in the supernatant of HTR-8/SVneo cells under different experimental conditions (Fig. 4).

We observed that uninfected cells and treated with meloxicam (10 μg/mL: ^*^P = 0.0245; 250 μg/mL: ^*^P = 0.0001) or celecoxib (1 μg/mL: ^*^P = 0.0204; 5 μg/mL: ^*^P = 0.0342) increased the MIF production when compared to untreated and uninfected cells (Fig. 4A). However, the presence of parasite and COX-2 inhibitors did not change the MIF levels when compared to untreated and infected cells (Fig. 4E).

In relation to IL-6, we observed that uninfected cells and treated with higher concentration of meloxicam (^*^P = 0.0241) or celecoxib (^*^P = 0.0004) increased the production of this cytokine when compared to untreated and uninfected cells (Fig. 4B). Untreated and infected cells not changed IL-6 levels, but infected cells and treated with both concentrations of meloxicam (10 μg/mL: ^*^P = 0.0110; 250 μg/mL: ^*^P = 0.0222) or with 5 μg/mL of celecoxib (^*^P < 0.0001) increased the IL-6 production when compared to untreated and uninfected cells (Fig. 4F). Furthermore, infected cells and treated with higher concentration of meloxicam (^#^*P* = 0.031) or celecoxib (^#^*P* < 0.0001) increased the IL-6 production when compared to untreated and infected cells, and celecoxib showed a dose-dependent effect (Fig. 4F).

Regarding to IL-4, uninfected cells and treated with both concentrations of meloxicam (10 μg/mL: ^*^P = 0.003; 250 μg/mL: ^*^P = 0.0147) and 1 μg/mL of celecoxib (^*^P = 0.0191) increased the production of this cytokine when compared to untreated and uninfected cells (Fig. 4C). However, infected cells and treated with both concentrations of meloxicam (10 μg/mL: ^#^*P* = 0.0141; 250 μg/mL: ^#^*P* = 0.0057) or celecoxib (1 μg/mL: ^#^*P* = 0.0027; 5 μg/mL: ^#^*P* = 0.0015) decreased the IL-4 production when compared to untreated and infected cells (Fig. 4G).

In relation to TGF-β1, we observed that uninfected cells and treated with both concentrations of meloxicam increased the production of this cytokine when compared to untreated and uninfected cells (10 μg/mL: ^*^P < 0.0001; 250 μg/mL: ^*^P = 0.0005) (Fig. 4D). However, infected cells and treated with both concentrations of meloxicam (10 and 250 μg/mL: ^#^*P* < 0.0001) or celecoxib (1 and 5 μg/mL: ^#^*P* < 0.0001) decreased TGF-β1 levels when compared to untreated and infected cells, with meloxicam presenting a dose-dependent effect (Fig. 4H).

Moreover, we observed the nitrite production in these cells (Supplementary Fig. S1). We observed that uninfected cells and treated with 10 μg/mL of meloxicam or both concentrations of celecoxib decreased the nitrite production when compared to untreated and uninfected cells (^*^P < 0.0001), except the concentration 250 μg/mL of meloxicam (Supplementary Fig. S1A). However, infected cells and treated with both concentrations of meloxicam (10 μg/mL: ^#^*P* = 0.0203; 250 μg/mL: ^#^*P* < 0.0001) or celecoxib (1 and 5 μg/mL: ^#^*P* < 0.0001) increased the nitrite production when compared to untreated and infected cells, with meloxicam presenting a dose-dependent effect (Supplementary Fig. S1B).

In summary, our data show that COX-2 inhibitors increase IL-6 and nitrite, and decrease IL-4 and TGF-β1 production in HTR-8/SVneo cells, triggering a pro-inflammatory profile in HTR-8/SVneo cells.

IL-8, IL-10 and TNF-α showed values below detection levels in all conditions tested in HTR-8/SVneo cells (data not shown).

### *T. gondii* increased lipid droplets formation in BeWo and HTR-8/SVneo cells

**Figure 5:**
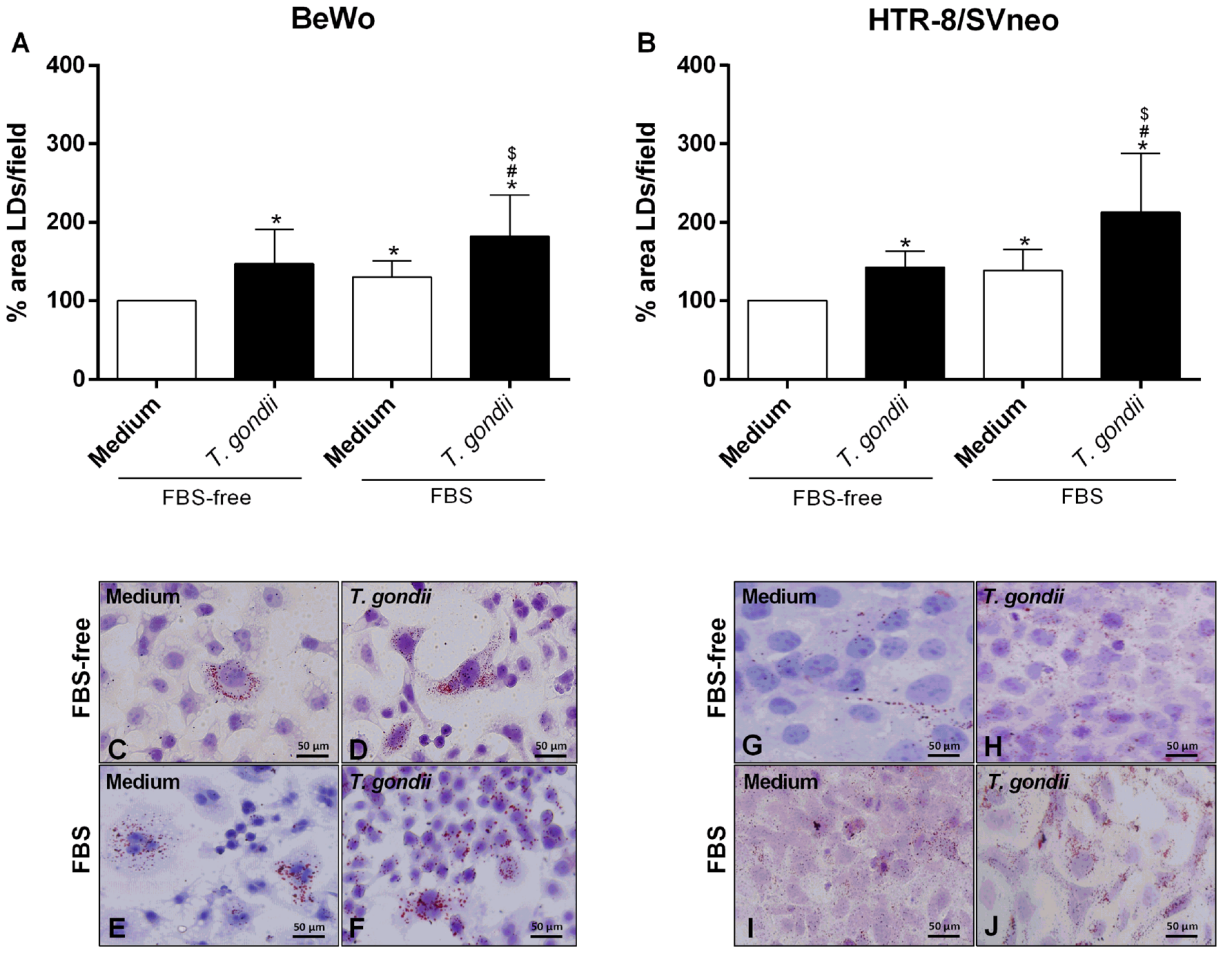
LDs production in BeWo and HTR-8/SVneo cell infected with *T. gondii*. BeWo (**A**) and HTR-8/SVneo (**B**) cells were infected or not with *T. gondii* in absence or presence of FBS for 24 h. Next, cells were stained with Oil Red O (0.5%) for visualization of lipid droplets (LDs), and 40 images from differents fields for each condition were analyzed by Image J software version 1.50i (National Institutes of Health, USA, https://imagej.nih.gov/ij). Data were presented as percentage (%) of area LDs/field in relation to uninfected cells FBS-free (100% area LDs). Data were shown as mean ± SEM from three independents experiments with four replicates. Differences between groups were analyzed by Kruskal–Wallis test and Dunn’s multiple comparison post-test (GraphPad Prism Sofware version 6.01, https://www.graphpad.com). Significant differences in relation to medium FBS-free (^*^), medium with FBS (^#^) or *T. gondii* FBS-free (^$^). Differences were considered significant when *P* < 0.05. Representative photomicrographs are shown in BeWo (**C**–**F**) and HTR-8/SVneo cells (**G**–**J**) uninfected (**C**, **G)** or infected (**D**, **H**) and FBS-free, and uninfected (**E**, **I**) or infected (**F**, **J**) with FBS. Staining by Oil Red O and Harris-hematoxylin. Bar scale: 50.0 µm.

After evaluating the parasite proliferation and immune response in BeWo and HTR-8/SVneo cells infected and treated with COX-2 inhibitors, we evaluated the LDs formation in these cells infected or not with *T. gondii* (Fig. 5).

Previous studies demonstrated that murine peritoneal macrophages increased the LDs production when treated with mouse serum, decreasing the microbicidal capacity against *T. gondii*^45^. Then, in a firts step of experiments, we evaluated the effect of fetal bovine serum (FBS) in LDs production in uninfected and infected cells for 24 h. Uninfected or infected BeWo and HTR-8/SVneo cells, in presence of FBS, increased the LDs production when compared to uninfected cells FBS-free (^*^P = 0.0012; ^*^P = 0.0243, respectively) or infected cells FBS-free (^$^*P* < 0.0001) (Fig. 5A,B).

In a second step of experiments, we verified the effect of *T. gondii* in the LDs production. In BeWo (Fig. 5A) and HTR-8/SVneo (Fig. 5B) cells, the infection in absence (^*^P < 0.0001; ^*^P = 0.0006, respectively) or presence (^#^*P* < 0.0001) of FBS increased the LDs production when compared to respective control (^*^medium FBS-free and ^#^medium with FBS, respectively). Representative photomicrographs are shown in BeWo (Fig. 5C–F) and HTR-8/SVneo cells (Fig. 5G–J). Uninfected or infected and FBS-free (Fig. 5C,D,G,H), and uninfected or infected with FBS (Fig. 5E,F,I,J).

For confirm the effect of *T. gondii* infection in LDs production in absence of FBS, we also evaluated the production of these organelles by confocal microscopy (Supplementary Fig. S2). Our data confirmed that the infection induced the LDs production in BeWo (^*^P = 0.0003, Supplementary Fig. S2A) and HTR-8/SVneo cells (^*^P = 0.0405, Supplementary Fig. S2B) in comparison to untreated cells (medium). Representative photomicrographs are shown in BeWo (Supplementary Fig. S2C) and HTR-8/SVneo cells (Supplementary Fig. S2D).

Thus, the infection and the presence of FBS induced LDs production. For this reason, all experiments were performed in the absence of FBS from the moment of infection.

### Lipid droplets formation decreased in BeWo and HTR-8/SVneo cells infected and treated with COX-2 inhibitors

**Figure 6:**
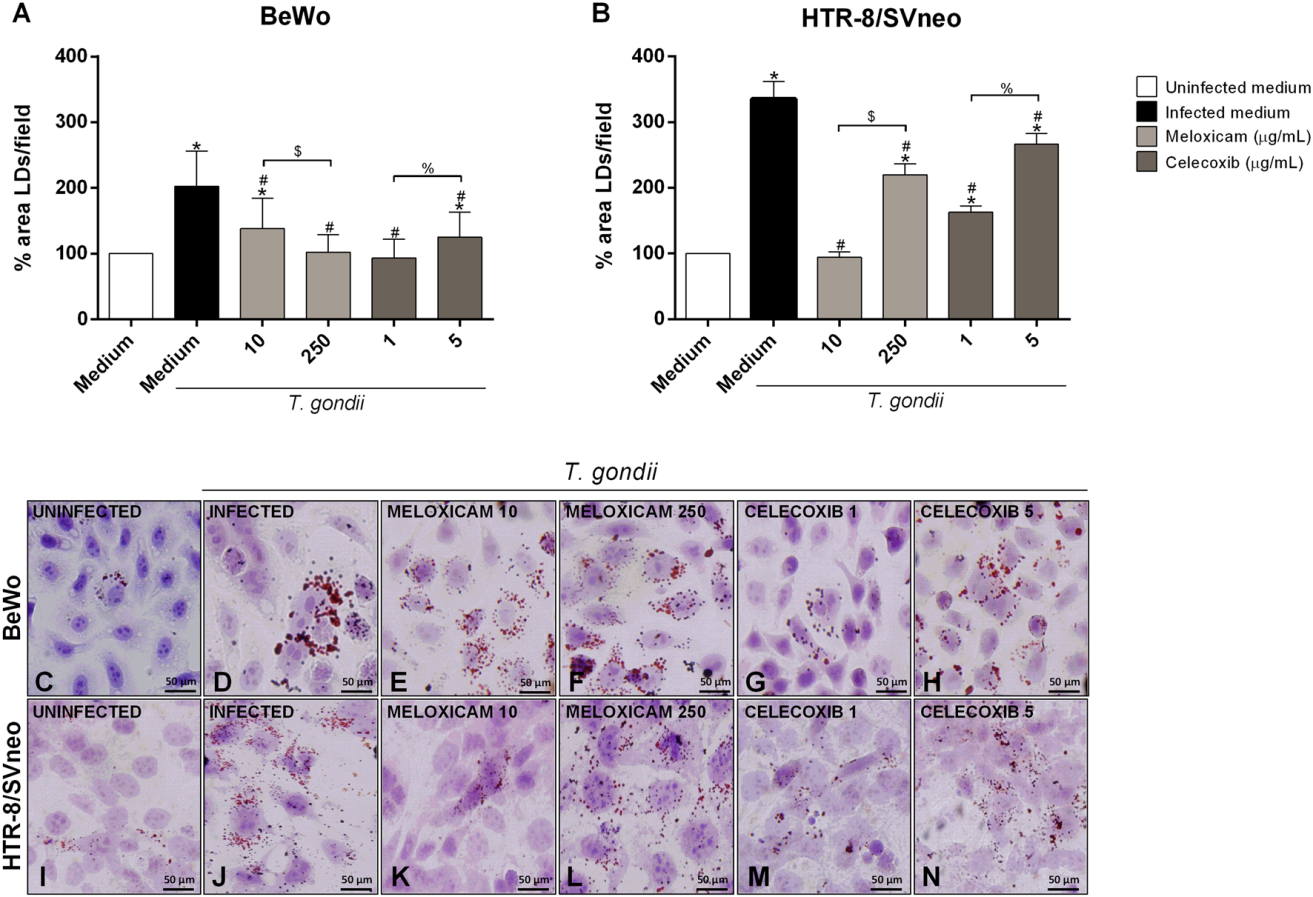
LDs production in BeWo and HTR-8/SVneo cells infected with *T. gondii* and treated with COX-2 inhibitors. BeWo (**A**) and HTR-8/SVneo (**B**) cells were infected or not with *T. gondii* for 3 h and treated or not with meloxicam or celecoxib for an additional 24 h. Next, cells were stained with Oil Red O (0.5%) for visualization of LDs, and 40 images from differents fields for each condition were analyzed by the Image J software version 1.50i (National Institutes of Health, USA, https://imagej.nih.gov/ij). Data were presented as percentage (%) of area LDs/field in relation to uninfected and untreated cells (100% area LDs). Data were shown as mean ± SEM from three independents experiments with four replicates. Differences between groups were analyzed by Kruskal–Wallis test and Dunn’s multiple comparison post-test (GraphPad Prism Sofware version 6.01, https://www.graphpad.com). Significant differences in relation to untreated and uninfected cells (^*^uninfected medium), untreated and infected cells (^#^infected medium), and between concentrations of meloxicam (^$^) or celecoxib (^%^). Differences were considered significant when *P* < 0.05. Representative photomicrographs are shown in BeWo (**C**–**H**) and HTR-8/SVneo cells (**I**–**N**) uninfected and untreated (**C**, **I**), infected and untreated (**D**, **J**), infected and treated with 10 μg/mL meloxicam (**E**, **K**), or 250 μg/mL (**F**, **L**), or infected and treated with 1 μg/mL celecoxib (**G**, **M**), or 5 μg/mL (**H**, **N**). Staining by Oil Red O and Harris-hematoxylin. Bar scale: 50.0 µm.

Next, we evaluated the LDs production under different experimental conditions with COX-2 inhibitors (Fig. 6). In BeWo cells, we observed that untreated and infected cells (^*^P < 0.0001) induced an increase in LDs production when compared to untreated and uninfected cells (Fig. 6A). On the other hand, the treatment of infected cells with both concentrations of meloxicam (^#^*P* < 0.0001) or celecoxib (1 μg/mL: ^#^*P* < 0.0001; 5 μg/mL: ^#^*P* = 0.0041) reduced significantly the LDs production in comparison to untreated/infected cells (Fig. 6A). The concentrations 250 μg/mL of meloxicam (^$^*P* = 0.0009) and 1 μg/mL of celecoxib (^%^*P* < 0.0001) reduced more the LDs production, showing a dose-dependent effect (Fig. 6A).

In HTR-8/SVneo cells, we observed that untreated and infected cells (^*^P < 0.0001) increased the LDs production when compared to untreated and uninfected cells (Fig. 6B). In contrast, the treatment of infected cells with both concentrations of meloxicam (10 μg/mL: ^#^*P* < 0.0001; 250 μg/mL: ^#^*P* = 0.0134) or celecoxib (1 μg/mL: ^#^*P* = 0.0008; 5 μg/mL: ^#^*P* = 0.0295) decreased the LDs production when compared to untreated/infected cells, which it is possible to observe that the lower concentration of meloxicam (^$^*P* < 0.0001) or celecoxib (^%^*P* = 0.0491) reduced LDs production in a dose-dependent manner (Fig. 6B).

Representative photomicrographs are shown in BeWo (Fig. 6C–H) and HTR-8/SVneo cells (Fig. 6I–N). Uninfected and untreated (Fig. 6C,I), infected and untreated (Fig. 6D,J), infected and treated with meloxicam 10 μg/mL (Fig. 6E,K), 250 μg/mL (Fig. 6F,L), or infected and treated with celecoxib 1 μg/mL (Fig. 6G,M), 5 μg/mL (Fig. 6H,N). Thus, the COX-2 inhibiton decreased the LDs production in both cells infected by *T. gondii*.

### COX-2 inhibitors were not toxic and reduced the *T. gondii* intracellular proliferation in human villous explants

**Figure 7:**
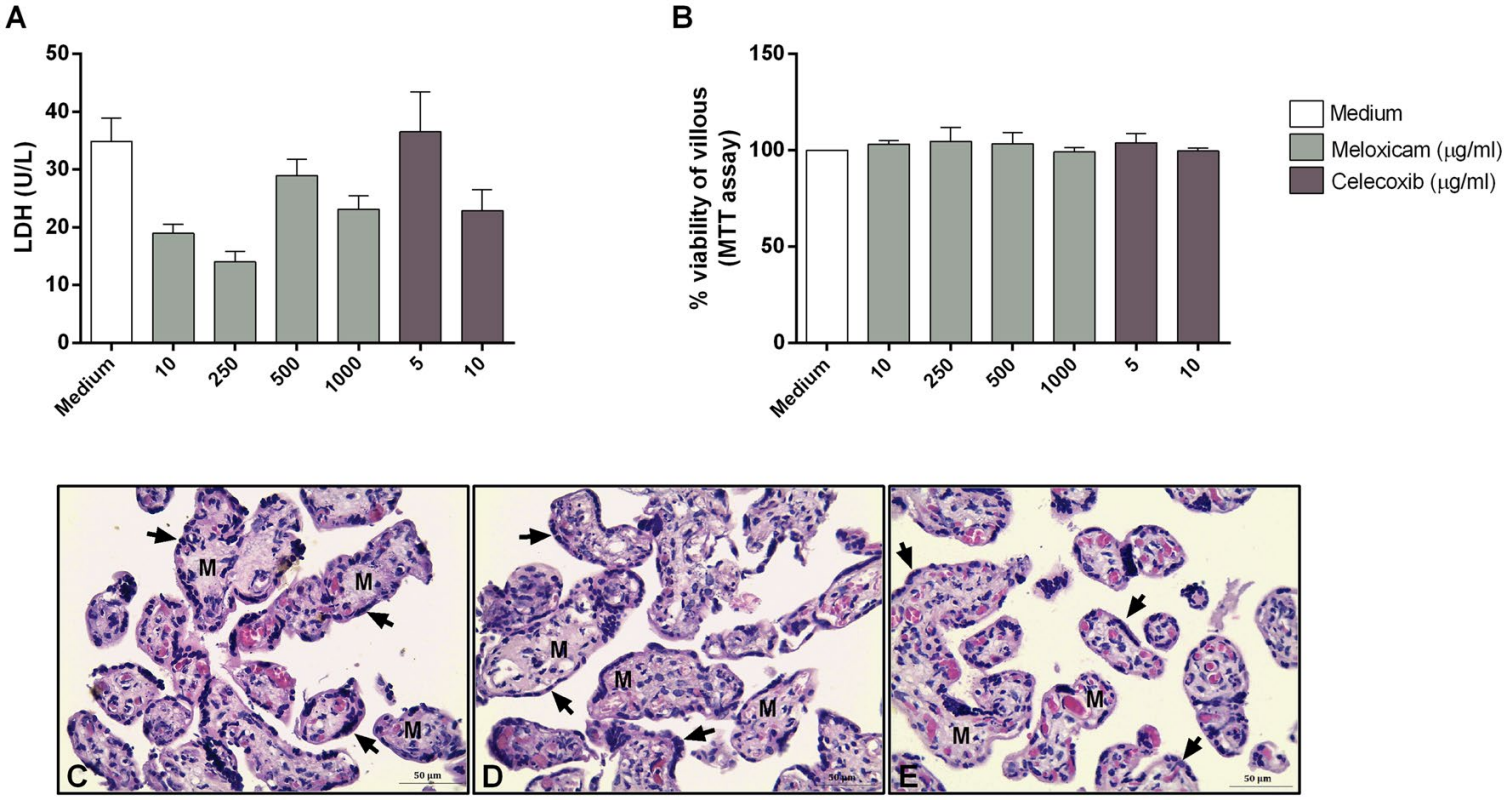
Viability of human villous explants treated with COX-2 inhibitors. Villous explants were treated with meloxicam or celecoxib for 24 h. Next, the supernatants were collected and lactate dehydrogenase (LDH) activity was measured (**A**) and villous explants were analyzed by MTT assay (**B**), and tissue viability was expressed in percentage (%) of villous viability in relation to untreated villous (medium, 100% of villous viability). Data were shown as mean ± SEM from two independents experiments with eight replicates. Differences between groups were analyzed by One-Way ANOVA test with Sidak’s multiple comparison post-test (**A**) or Kruskal–Wallis test and Dunn’s multiple comparison post-test (**B**) (GraphPad Prism Sofware version 6.01, https://www.graphpad.com). Representative photomicrographs of untreated villous explants (**C**), or treated with meloxicam (**D**) or celecoxib (**E**). Histological sections stained by Harris-hematoxylin and eosin. Arrows indicate the syncytiotrophoblast layers and M indicate the mesenchyme. Bar scale: 50.0 µm.

After evaluating the effects of COX-2 inhibitors in in vitro models, we evaluated the influence of COX-2 inhibitors in human villous explants, an ex vivo model of maternal–fetal interface (Fig. 7).

Firstly, based on the measurement of the LDH enzyme, we observed that no concentration of both inhibitors changed the villous viability (Fig. 7A). Additionally, we performed the MTT assay and observed the same effect (Fig. 7B). Based on these data, we chosed the concentration of 250 μg/mL meloxicam and 5 μg/mL celecoxib for further ex vivo experiments. Representative photomicrographs of untreated villous (Fig. 7C), or villous treated with 250 μg/mL meloxicam (Fig. 7D) or 5 μg/mL celecoxib (Fig. 7E), where it is possible to observe the morphology of the syncytiotrophoblast (arrow) and mesenchyme (M) preserved when compared to untreated villous (medium).

**Figure 8:**
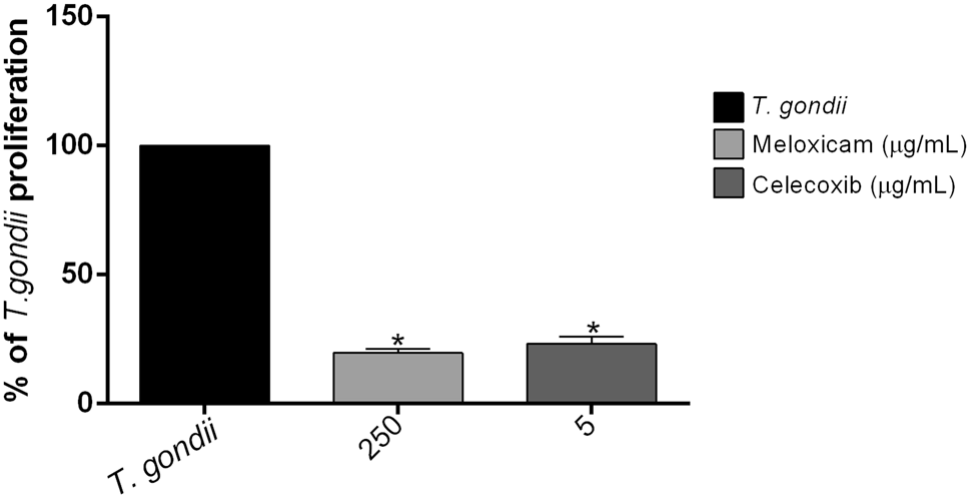
*T. gondii* intracellular proliferation in human villous explants treated with COX-2 inhibitors. Villous explants were infected with *T. gondii* for 24 h, and treated with meloxicam or celecoxib for an additional 24 h. Next, the villous explants were macerated for β-galactosidase assay, and data were presented as percentage (%) of *T. gondii* proliferation in relation to untreated and infected villous (100% of intracellular proliferation). Data were shown as mean ± SEM from two independents experiments with eight replicates. Differences between groups were analyzed by Kruskal–Wallis test and Dunn’s multiple comparison post-test (GraphPad Prism Sofware version 6.01, https://www.graphpad.com). Significant differences in relation to untreated and infected villous (^*^T. gondii). Differences were considered significant when *P* < 0.05.

Next, we evaluated the effect of COX-2 inhibitors in *T. gondii* intracellular proliferation. We observed that meloxicam (250 μg/mL: ^*^P = 0.0030) and celecoxib (5 μg/mL: ^*^P = 0.0043) decreased the parasite replication when compared to untreated villous (*T. gondii*) (Fig. 8).

### COX-2 inhibitors increased MIF and decreased TNF-α and IL-10 in human villous explants infected by *T. gondii*

**Figure 9:**
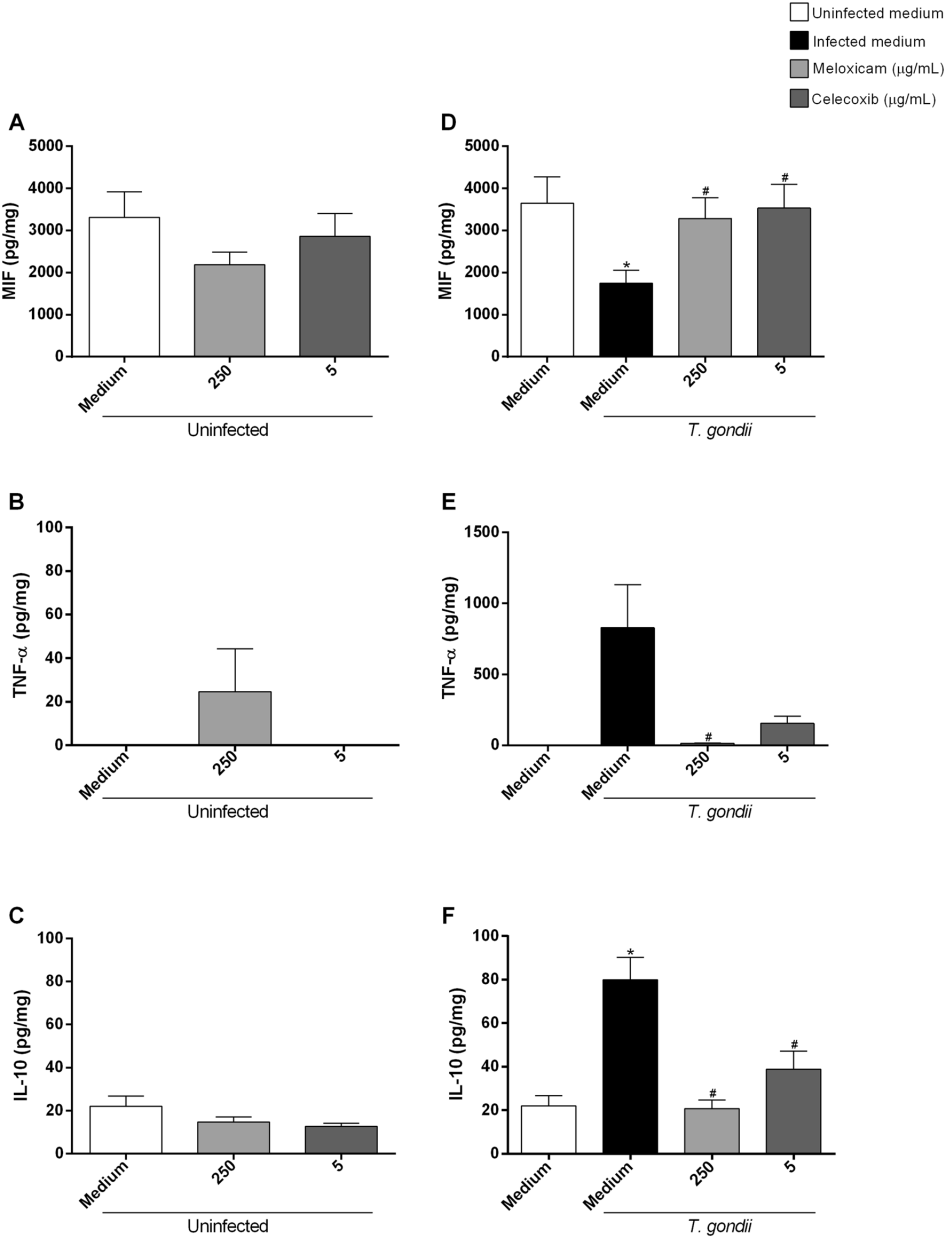
Cytokines production in human villous explants infected and treated with COX-2 inhibitors. Villous explants were infected or not with *T. gondii* for 24 h and treated or not with meloxicam or celecoxib for an additional 24 h. Next, the supernatants were collected for measurement of MIF (**A**, **D**), TNF-α (**B**, **E**) and IL-10 (**C**, **F**) by ELISA. Data were shown as mean ± SEM from two independents experiments with eight replicates. Differences between groups were analyzed by One-Way ANOVA test with Sidak’s multiple comparison post-test (**A**, **D**, **C**, **F**) or Kruskal–Wallis test and Dunn’s multiple comparison post-test (**B**, **E**) (GraphPad Prism Sofware version 6.01, https://www.graphpad.com). Significant differences in relation to untreated and uninfected villous (^*^uninfected medium) and untreated and infected villous (^#^infected medium). Differences were considered significant when *P* < 0.05.

Next, we evaluated the cytokine profile in supernatant of untreated and uninfected, treated and uninfected, untreated and infected, and infected and treated villous with meloxicam or celecoxib (Fig. 9).

We observed that uninfected villous and treated with both inhibitors not induced MIF production when compared to untreated and uninfected villous (Fig. 9A). In untreated and infected villous, the production of this cytokine decreased when compared to untreated and uninfected villous (^*^P = 0.0094) (Fig. 9D). However, infected villous and treated with meloxicam (^#^*P* = 0.0238) or celecoxib (^#^*P* = 0.0098) increased the MIF production when compared to untreated and infected villous (Fig. 9D).

In relation the TNF-α production, we observed that only uninfected villous and treated with meloxicam produced this cytokine (Fig. 9B). In untreated and infected villous, the TNF-α production increased, but infected villous and treated with meloxicam decreased the production of this cytokine when compared to untreated and infected villous (^#^*P* = 0.0445) (Fig. 9E).

Regarding to IL-10 production, we observed that uninfected villous and treated with both COX-2 inhibitors did not alter the production of this cytokine when compared to untreated and uninfected villous (Fig. 9C). In untreated and infected villous, the IL-10 production increased when compared to untreated and uninfected villous (^*^P < 0.0001). However, infected villous and treated with meloxicam (^#^*P* < 0.0001) or celecoxib (^#^*P* = 0.0007) decreased the production of this cytokine when compared to untreated and infected villous (Fig. 9F).

In summary, our data show that COX-2 inhibitors increase MIF and decrease TNF-α and IL-10 production in human villous explants.

IL-4, IL-8 and TGF-β1 showed values below detection levels in all conditions tested (data not shown).

### Proposed model for the action of COX-2 inhibitors during *T. gondii* infection in human trophoblast cells and human villous explants

**Figure 10:**
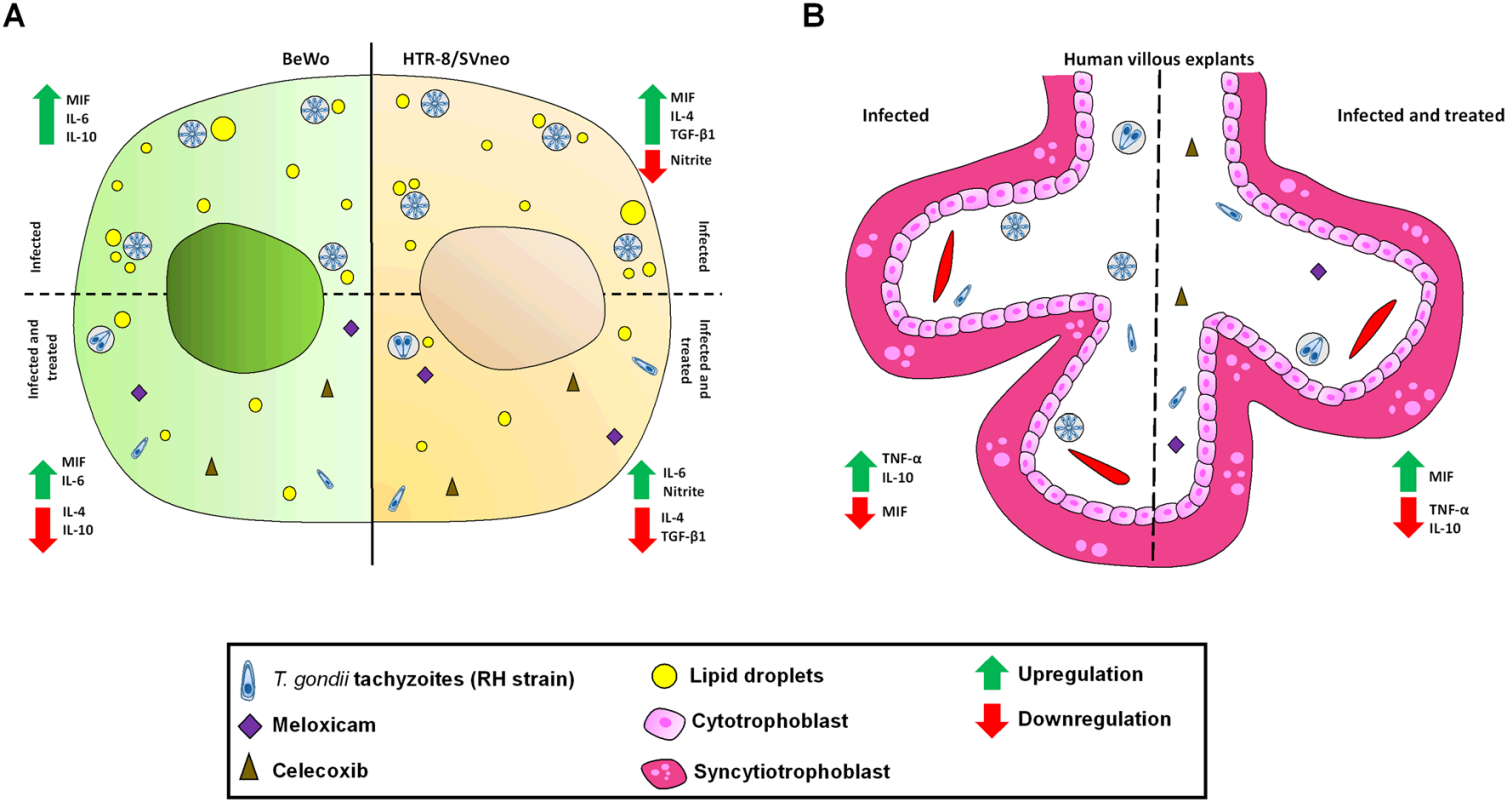
Proposed model of the role COX-2 inhibitors in human trophoblast cells and human villous explants. (**A**) BeWo cells (left panel) infected with *T. gondii* and treated with COX-2 inhibitors reduced parasite intracellular proliferation, upregulated pro-inflammatory cytokines (MIF and IL-6) e downmodulated anti-inflammatory cytokines (IL-4 and IL-10), besides decreasing the LDs production. In addition, HTR-8/SVneo cells (right panel) infected with *T. gondii* and treated with COX-2 inhibitors also reduced parasite intracellular proliferation, upregulated MIF and nitrite and downmodulated IL-4 and IL-10, besides also decreasing the LDs production. (**B**) Human villous explants infected with *T. gondii* and treated with COX-2 inhibitors reduced *T. gondii* intracellular proliferation, upregulated MIF and downmodulated TNF-α and IL-10.

Based on our results, a proposed model of the actions induced by COX-2 inhibitors in in vivo and ex vivo experimental models is shown in Fig. 10. When BeWo cells were infected with *T. gondii* and treated with COX-2 inhibitors, there was a reduced parasite intracellular proliferation, upregulation of pro-inflammatory cytokines (MIF and IL-6), downmodulation of anti-inflammatory cytokines (IL-4 and IL-10), and a decrease in LDs production (Fig. 10A, left panel). In addition, HTR-8/SVneo cells infected with *T. gondii* and treated with COX-2 inhibitors also reduced parasite intracellular proliferation, upregulated IL-6 and nitrite, downmodulated IL-4 and TGF-β1, and also reduced the LDs production (Fig. 10A, right panel). When we evaluated the role of COX-2 in ex vivo model, we observed that human villous explants infected with *T. gondii* and treated with COX-2 inhibitors reduced *T. gondii* intracellular proliferation, upregulated MIF and downmodulated TNF and IL-10 (Fig. 10B). Thus, COX-2 is an important inflammatory mediator for the *T. gondii* proliferation, since its inhibition induced a pro-inflammatory response and decreased the production of LDs, favoring the control of the parasite growth.

## Discussion

COX-2 and PGE_2_ can be stored and produced in lipid droplets^63^, and studies show that many pathogens are able to increase the production of these organelles to evade the host immune response^39,64^. However, the role of COX-2 in *T. gondii* infection, as well as its importance in LDs production is not well understood in many cells type, including in maternal–fetal interface. Thus, in this work we evaluated the functional role of COX-2 during infection by *T. gondii* (RH strain, clone 2F1) and its effect on the LDs production in human trophoblastic cells.

Firstly, we analyzed the possible cytotoxicity effect of COX-2 inhibitors (meloxicam and celecoxib) in BeWo and HTR-8/SVneo cells. We observed that both inhibitors did not decrease the viability of both cell lines. These results are in agreement with studies by our group, where the same concentrations of both inhibitors did not alter the viability of peritoneal macrophages^53^. In other study using aspirin and celecoxib as COX-2 inhibitors, they observed that the concentrations used also did not alter the viability of cardiac myoblast cells^65^.

Next, we analyze whether COX-2 inhibitors would modulate the *T. gondii* intracelular proliferation. Our results demonstrated that both inhibitors were able to decrease parasitism in dose-dependent manner in both cell types, suggesting that susceptibility to *T. gondii* is modulated by COX-2 in villous and extravillous human trophoblast cells, and this effect is probably related to a decrease of PGE_2_. Our findings are in agreement with many other studies. Previous studies have demonstrated an association of COX-2/PGE_2_ in susceptibility in *Leishmania* spp. and *T. cruzi* infection. In macrophages coinfected with HIV-1 and *Leishmania amazonensis*, the TAT viral protein induced greater COX-2 expression and PGE_2_ production, favoring the growth of *L. amazonensis*, but this effect was reversed when COX-2 was inhibited by celecoxib^66^. Moreover, in murine mononuclear phagocyte derived from B-1 cells (B-1CDP) infected with *L. major,* the treatment with COX-2 inhibitors (aspirin, indomethacin or NS-398) decreased the parasites number^67^. Also, cells-enriched peripheral human blood cells population treated with celecoxib promoted a decrease in *T. cruzi* infection, however, when these cells were treated with celecoxib plus PGE_2_, the rate of infected cells with *T. cruzi* was restored when compared to cells treated only with celecoxib, but aspirin (more selective for COX-1 than COX-2) did not present the same effect^68^. In contrast, Swiss mice infected with *T. cruzi* and treated with aspirin reduce parasitemia during an acute or chronic phases^69^. Recently, our research group demonstrated that *Calomys callosus* rodents, murine peritoneal macrophages and human monocytes infected by *T. gondii* (ME-49 or RH strains) and treated with COX-2 inhibitors (celecoxib or meloxicam) decreased parasitism^53^. However, when human monocytes were treated with COX-2 inhibitors plus PGE_2_, the parasitism was restored, highlighting the importance of these inflammatory mediators in *T. gondii* infection^53^. However, there are no studies about COX-2 and *T. gondii* in maternal–fetal interface of any specie, and it is widely established by scientific literature that maternal–fetal interface is an environment completely different in aspects about immune response, physiology and several other mechanisms^70^. Thus, our present study is innovator, since is the first showing the role of COX-2 in the susceptibility of *T. gondii* infection in human trophoblastic cells.

Then, we investigate some possible mechanisms that COX-2 inhibitors would be interfering in *T. gondii* infection. Studies have showed that PGE_2_ can inhibit the pro-inflammatory immune response (Th1 profile). Therefore, the COX-2 inhibitors and, consequently, the decrease of PGE_2_ production, could restore the Th1 profile, leading to parasitism control^52,71^. In this sense, we evaluated the cytokines profile secreted by human trophoblastic cells. In BeWo cells, both COX-2 inhibitors increased MIF and IL-6, and decreased IL-4 and IL-10, while in HTR-8/SVneo cells, both COX-2 inhibitors increased IL-6 and nitrite, and decreased IL-4 and TGF-β1. Although BeWo and HTR-8/SVneo cells presented a pro-inflammatory profile induced by COX-2 inhibitors, they demonstrated different expression of cytokines. Additonally, BeWo cells and HTR-8/SVneo showed below detection levels of IL-8, TGF-β1, and TNF, or IL-8, TNF and IL-10, respectively. Regarding to *T. gondii* infection in experimental models of maternal–fetal interface, previous studies also have reported, by ELISA assay, undetectable levels of TGF-β1, IL-12p70, TNF-α, and IFN-γ cytokines produced by BeWo cells, even after parasite infection^56,57^. In addition, a weak expression of IL-8 is commonly observed in unstimulated BeWo cells^72^.

Studies have shown that IL-6 is a pro-inflammatory cytokine due to its protective role against pathogens, such as *T. gondii*^13,54^. Furthermore, corroborating our findings, *C. callosus* rodents infected with *T. gondii* and treated with both COX-2 inhibitors also increased IL-6 production^53^, showing the importance of this cytokine in the parasitism control. BeWo and HTR-8/SVneo cells showed increase IL-6 when treated with COX-2 inhibitors, and it was an important mechanism shared by both population of trophoblast to control the parasite replication. Also, both cells secreted IL-6 in basal conditions. IL-6 is a multifunctional cytokine produced by human trophoblast, since is associated with normal placental development and successful pregnancy^73–75^. Therefore, it is plausible that BeWo and HTR-8/SVneo modulate similarly IL-6 to ensure adequate physiology of the maternal–fetal interface. MIF is another important pro-inflammatory cytokine in the immune response against pathogens^76,77^, and studies showed their protective role in *T. gondii* infection^14,15,53,56^. Although studies showing its protective role against *T. gondii*, including in BeWo cells^14,54,78^, recently a study by our group showed that inhibition of MIF decreases the *T. gondii* proliferation in HTR-8/SVneo cells^59^. Moreover, the addition of recombinant MIF increased COX-2 expression, favoring the *T. gondii* growth in HTR-8/SVneo cells^59^. Our findings in HTR-8/SVneo cells showed that COX-2 inhibitors did not increase MIF production when compared to only infected cells, but the inhibitors induced an upregulation of this cytokine in BeWo cells, suggesting that parasitism control in HTR-8/SVneo is not related to this cytokine. Thus, different population of human trophoblast can present difference expression of cytokines, as MIF, since villous trophoblast can control *T. gondii* infection using MIF, but extravillous trophoblast do not present the same ability.

In addition, our findings showed that infected HTR-8/SVneo cells decreased nitrite production, but when COX-2 was inhibited, its production increased, corroborating with other studies, where COX-2 inhibition also increased nitrite production^53,65^, showing the importance of this inflammatory mediator in infection control. On the other hand, nitrite production was not observed in BeWo cells. It is possible to speculate that nitrite is harmful for immune tolerance in placental environment (villous trophoblast), however previous study showed active role of nitrite in HTR-8/SVneo cells (extravillous trophoblast)^79^, once again evidencing that differences of inflammatory mediators between trophoblasts cells are normal and expected.

Although there is a strong immune response to control the infection, the parasite seeks mechanisms to evade the host immune response, such as the induction of anti-inflammatory cytokines. Studie by our group showed that the susceptibility to *T. gondii* infection in BeWo cells was increased by IL-10 and TGF-β1^80^. Our findings demonstrated that the infection by *T. gondii* induced the IL-4, IL-10 and TGF-β1 production, however, COX-2 inhibitors decreased these cytokines and also contributed to parasitism control. Corroborating our findings, studies showed that human monocytes infected with *T. gondii* and B-1CDP infected with *L. major* and treated with COX-2 inhibitors (meloxicam, celecoxib or aspirin) decreased the production of IL-10 when compared to only infected cells^53,67^. Moreover, spleen cells of BALB/c mice infected with *T. cruzi* and treated with COX-2 inhibitor (sodyum salicilate or meloxicam) decreased the IL-4 and IL-10 production^81^. Both BeWo and HTR-8/SVneo cells modulated IL-4, but they were different in relation to IL-10 and TGF-β1. Our previous study showed that HTR-8/SVneo is able to modulate TGF-β1, and we could not verify IL-10 in these cells^58^, although we demonstrated that BeWo can modulate TGF-β1 and IL-10^80^. We have to consider that, in the present study, both cell lines were submitted to COX-2 inhibitors, and this is the first study to demonstrate the role of this enzyme in maternal–fetal interface, thus is plausible that different population of trophoblast can express different cytokines.

It has been demonstrated that intracellular tachyzoites of *T. gondii* are able to upregulate the LDs production by host cells and recruit these organelles to close the parasitophore vacuole, which together favors the parasite replication^40,41^. Moreover, literature findings show that LDs are source of COX-2 and PGE_2_^82^ and that the presence of COX-2 and PGE_2_ in LDs may be associated with the success in the parasite proliferation^64^. Illustrating that, some pathogens modulate the expression of the COX-2/PGE_2_ axis to subvert the host immune response^39,64^. In this line of discussion, our data demonstrated that the COX-2 inhibition caused a reduction in the number of intracellular tachyzoites in both cell types and in villous explants. Thus, to understand the underlying mechanisms associated with the parasite control mediated by COX-2 inhibitors, besides immune response, we assessed the impact of the COX-2 inhibition in the LDs production in BeWo and HTR-8/SVneo cells infected by *T. gondii*. Interestingly, our findings showed that the COX-2 inhibition resulted in a decrease of LDs in both cell lines; thus, these data highlight that the blockage of the COX-2 activity reduce the availability of LDs to *T. gondii*, which could explain, in part, the impairment in the parasite replication. Thus, this is the first study to show the influence of COX-2 in LDs expression during *Toxoplasma* infection in maternal–fetal interface.

Lovo-Martins et al.^83^ showed that C57BL/6 mice treated with extracellular vesicles from *T. cruzi* of Y strain (EV Y) had increased cardiac parasitism and high internalization of the parasite in bone marrow-derived macrophages, and these effects were associated with the LDs formation and PGE_2_ production. Macrophages infected with *T. cruzi* increased the LDs production, leading to a higher PGE_2_ production and contributing to the growth of the parasite. However, the COX-2 inhibitor reversed this effect^35^, corroborating our findings. Thus, the parasitism control in our results may also be related to the decrease in LDs. However, our data showed that the lower concentrations of both inhibitors, except meloxicam in BeWo cells, were more effective in reducing the LDs production than the higher concentrations, different of data from parasite intracellular proliferation, where the higher concentrations of COX-2 inhibitors were more efficient in controlling parasitism. However, the dose-dependent effect of COX-2 inhibitors in controlling *T. gondii* infection in BeWo and HTR-8/SVneo cells can be explained by the dose-dependent effect of the inhibitors in inducing a strong pro-inflammatory immune response, such as upregulation of IL-6 and MIF. Thus, it is possible to suggest that the immune response is more efficient in controlling parasitism in human trophoblastic cells than the amount of LDs. Furthermore, since *T. gondii* can consume the content of these organelles, it is possible that in higher doses of COX-2 inhibitors, because there are fewer parasites, there was higher lipid droplets availability.

After evaluating the role of COX-2 inhibitors in in vitro models, we investigated the effect of these inhibitors in ex vivo model. We observed that both inhibitors did not alter the viability of human villous explants and were also able to strongly reduce the *T. gondii* intracellular proliferation. Studies demonstrate that the annexin A1 peptide was able to reduce parasitism in villous explants infected by *T. gondii*, and this was related to a decrease in COX-2/PGE_2_ production^84^. Regarding to cytokines production, we observed that only infected villous decreased the MIF production and increased IL-10, suggesting a possible escape mechanism for *T. gondii*, besides inducing a high production of TNF-α. However, both inhibitors reversed these effects, corroborating the parasitism findings. Our research group have demonstrated the protective role of MIF against *T. gondii* in human placenta^56,61,62,85^. The placental microenvironment is rich in IL-10, an anti-inflammatory cytokine that contributes to maternal–fetal tolerance, which can facilitate infection by *T. gondii*^80,86^, as shown in this study. Interestingly, COX-2 inhibitors decreased the production of this cytokine, contributing to the control of the parasitism. In addition, we also demonstrated that the infection of human villous by *T. gondii* induced a high TNF-α production, a pro-inflammatory abortogenic cytokine that can disrupt the maternal–fetal immune balance and cause miscarriage^87^. In contrast, COX-2 inhibitors decreased the TNF-α release, which may be important for the maintenance of pregnancy. Literature findings have demonstrated a relationship between TNF-α and COX-2^88–90^. Illustrating that, it was reported that TNF-α is capable of stimulating the COX-2 activation resulting in an augmentation of PGE_2_ by first trimester trophoblasts; surprinsigly, this phenomenon was reversed by the use of COX-2-selective inhibitors^88^. In this sense, combining our findings with literature, we hypothesized that the reduction in TNF-α levels, as result of COX-2 inhibition, may culminate in a decrease of PGE_2_ production by infected villous, which could explain the impairment of the parasite growth. Taken together, in agreement with published studies, our data reveal a different cytokine profile between human trophoblast cells and villous explants from human third trimester pregnancy in the context of *T. gondii*^56,57,60^. In this context, we suggested that these differences among both models may be explained, in part, due to the fact that human villous is a tissue composed of mesenchyme, villous and extravillous trophoblast and syncytiotrophoblast, culminating in a distinct cytokine production.

In conclusion, we demonstrated that COX-2 inhibitors impair *T. gondii* intracellular proliferation in human trophoblastic cells and human chorionic villous, since upregulates pro-inflammatory mediators and reduce the lipid droplets production in cells, dampening the environment for parasite survival. In this sense, the present findings are novel and highly essential in the context of placental environment.

However, future studies are necessary to clarify specific mechanisms involved, as signaling pathways triggered by modulated cytokines and associated to lipid droplets production in trophoblast cells. Thus, COX-2 may be a future therapeutic target for the treatment of congenital toxoplasmosis.

## Methods

### Cell culture

Human villous trophoblast cells (BeWo line) were obtained from American Type Culture Collection (ATCC, Manassas, VA, USA). The human extravillous trophoblast cells (HTR-8/SVneo line) were obtained from villous explants at early pregnancy and was a gift from Dr. Estela Bevilacqua (University of São Paulo, SP, Brazil). Both cells were cultured in culture flasks of 75 cm^2^ in RPMI 1640 medium (Cultilab, Campinas, SP, Brazil) supplemented with 100 U/mL penicillin (Sigma Chemical Co., St. Louis, MO, USA), 100 µg/mL streptomycin (Sigma) and 10% fetal bovine serum (FBS) (Cultilab) in a humidified incubator at 37 °C and 5% CO_2_^54,56^. The Ethics Committee of the Federal University of Uberlândia, MG, Brazil, communicates that studies performed with cell lines acquired commercially do not need ethical approval (Protocol #13/2012).

### Human villous explants culture

Third-trimester human placentas (36 to 40 weeks of pregnancy, n = 2) were acquired from pregnant patients after elective cesarean section deliveries at the Clinics Hospital of the Federal University of Uberlândia (HC-UFU), MG, Brazil. Exclusion criteria included pre-eclampsia, hypertension, infectious disease such as toxoplasmosis, chronic renal disease, cardiac disease, connective tissue disease, diabetes and other manifestations which could interfere with the results of this study. After being collected, placental tissues were washed in sterile phosphate-buffered saline (PBS, pH 7.2) to remove the excess of blood, and the dissection of the villous was performed using a stereomicroscope to remove endometrial tissue and fetal membranes up to 1 h after collection. Then, floating terminal chorionic villi containing five to seven free tips per explant were collected, placed in 96-well plates (one per well) and cultured in 200 µL RPMI 1640 medium with 10% FBS, penicillin (100 U/mL) and streptomycin (100 μg/ml) for 24 h at 37 °C and 5% CO_2_ for future experiments. The volume of the villous explants was ∼ 10 mm^3^ ^56,91^.

### Parasites

Tachyzoites of *T. gondii* (2F1 clone) were provided from Dr. Vern Carruthers (Medical School of Michigan University, Ann Arbor, MI, USA). This clone is derived from highly virulent RH strain and express the β-galactosidase gene, which enable a colorimetric assay to measure the parasites in cells or tissues^54,56^. These parasites were propagated in BeWo cells maintained in RPMI 1640 medium supplemented with penicillin (100 U/mL) and streptomycin (100 μg/ml) and 2% FBS at 37 °C and 5% CO_2_.

### Viability assay in BeWo and HTR-8/SVneo cells after COX-2 inhibitors treatment

BeWo (3 × 10^4^ cells/200 µL in 96-well plates) and HTR-8/SVneo (2 × 10^4^ cells/200 µL in 96-well plates) were treated with increasing concentrations of a preferential COX-2 inhibitor, meloxicam (Eurofarma Laboratórios, São Paulo, SP, Brazil) (1, 5, 10, 250 and 500 µg/mL), or a specific COX-2 inhibitor, celecoxib (Pfizer Pharmaceuticals LLC, Guarulhos, SP, Brazil) (1, 5 and 10 µg/mL) for 24 h in RPMI medium supplemented with penicilin, streptomycin and FBS-free in a humidified incubator at 37 °C and 5% CO_2_. COX-2 inhibitors concentrations were based on a previous study by our research group^53^. Meloxicam was diluted in RPMI medium FBS-free. However, celecoxib was dissolved in RPMI medium containing dimethyl sulfoxide (DMSO) in order to improve the dilution. To verify whether DMSO could be toxic to BeWo and HTR-8/SVneo cells, we treated the cells with 0.005% DMSO in RPMI medium FBS-free, the percentage used in the treatments with celecoxib. As control, cells were treated with only medium FBS-free (medium).

After 24 h of treatment, supernatants were collected and frozen at -80 °C for later measurements of cytokines and nitrite. In parallel, the 3-(4,5-Dimethyl-2-thiazolyl)-2,5-diphenyl2H-tetrazolium bromide (MTT, Sigma) assay was performed on the cells^92^. Cells were incubated for 4 h with 10 µL of MTT (5 mg/mL) plus 90 µL medium with 10% FBS in the same culture condition at 37 °C and 5% CO_2_. Formazan crystals were solubilized by adding a solution containing 10% sodium dodecyl sulfate (SDS, Sigma) and 50% N,N-dimethyl formamide (Sigma) for 30 min and the optical densities were measured at 570 nm (Titertek Multiskan Plus, Flow Laboratories, McLean, VA, USA). Data were expressed as percentage of viable cells (% cellular viability) in comparison to untreated cells (100% of cellular viability). Three independent experiments with eight replicates were performed.

### *T. gondii* intracellular proliferation in BeWo and HTR-8/SVneo cells after COX-2 inhibitors treatment

After verifying the toxicity of meloxicam or celecoxib by MTT, we chose two concentrations of both inhibitors to analyze *T. gondii* intracellular proliferation in BeWo and HTR-8/SVneo cells. BeWo (3 × 10^4^ cells/200 µL in 96-well plates) and HTR-8/SVneo (2 × 10^4^ cells/200 µL in 96-well plates) were infected with *T. gondii* tachyzoites in a proportion of one parasite per cell (1:1) in medium FBS-free, and after 3 h of culture, infected cells were treated with 10 and 250 µg/mL meloxicam or 1 and 5 µg/mL celecoxib in medium FBS-free for an additional 24 h. As controls, cells were infected and untreated (*T. gondii*). After 24 h, supernatants were collected and stored at -80 °C for later measurements of cytokines and nitrite. In parallel, *T. gondii* intracellular proliferation assay was performed in infected cells by β-galactosidase colorimetric assay, as previously describe^54^. *T. gondii* intracellular proliferation was obtained according to a reference curve containing free tachyzoites (from 1 × 10^6^ to 15.6 × 10^3^). The data were expressed as a percentage (%) of *T. gondii* proliferation: the mean number of tachyzoites from infected and untreated (*T. gondii*) corresponded to 100% parasite proliferation, then the number of tachyzoites from each treatment condition was transformed in percentage according to 100% of parasite proliferation from the control (*T. gondii*)^56^. The half-maximum antiparasitic activity (IC_50_) were calculated by extrapolation of the corresponding dose–response curve on a log-linear plot employing the portions of the curve that transected the 50% response point; the values were expressed in μg/mL ± standard desviation (SD)^93^. Three independent experiments in eight replicates were performed.

### Lipid droplets staining

In order to evaluate the production of lipid droplets in BeWo and HTR-8/SVneo cells, we performed Oil Red O (Sigma) staining^94^.

In the first step of the experimets, BeWo and HTR-8/SVneo cells were cultured on 13 mm glass coverslips (Sigma) (5 × 10^4^ cells/200 µL in 24-well plates), infected with *T. gondii* tachyzoites (1:1) in medium in absence or presence of FBS for 3 h, and then treated with medium in absence or presence of FBS for adittional 24 h. As control, cells were uninfected and maintained in medium FBS-free. In a second step, BeWo and HTR-8/SVneo cells were cultured on 13 mm glass coverslips (5 × 10^4^ cells/200 µL in 24-well plates), infected with *T. gondii* tachyzoites (1:1) in medium FBS-free and, after 3 h of culture, cells were treated with 10 and 250 µg/mL meloxicam or 1 and 5 µg/mL celecoxib in medium FBS-free for an additional 24 h. As controls, cells were uninfected and untreated (uninfected medium) or infected and untreated (infected medium) in medium FBS-free. After 24 h, cells from all experimental conditions were processed for analysis of lipid droplets. The glass coverslips with adherent cells were washed with sterile PBS, fixed with formaldehyde 3.7% in HBSS (pH 7.4) for 15 min, incubated with propylene glycol (100%) for 5 min, and stained with 0.5% Oil Red O (Sigma) diluted in 100% propylene glycol for 10 min at 60 °C. Next, cells were washed with 85% propylene glycol for 5 min and washed thrice with distilled water. In addition, as representative images, one glass coverslip from each condition was counter stained with Harris-hematoxylin for 30 s. The glass coverslips stained only with Oil Red O were analyzed under a light microscope (BX40, Olympus, Tokyo, Japan) (40 images from different fields of each condition). Images were analyzed in Image J software version 1.50i (National Institutes of Health, USA, https://imagej.nih.gov/ij). The data were expressed as the percentage of LDs (% area LDs/field) in each condition in comparison to untreated and uninfected cells (100% of LDs area in the field). Three independent experiments in four replicates were performed.

### Immunofluorescence

In addition, also to evaluate the production of lipid droplets in BeWo and HTR-8/SVneo cells infected, we performed confocal fluorescence microscopy assay.

BeWo and HTR-8/SVneo cells were cultured on 13 mm glass coverslips (Sigma) (5 × 10^4^ cells/200 µL in 24-well plates), infected with *T. gondii* tachyzoites (1:1) in medium FBS-free for 24 h. As control, cells were uninfected and maintained in medium FBS-free (medium). After, the glass coverslips with adherent cells were washed with sterile PBS, fixed with formaldehyde 10% for 10 min, washed twice with sterile PBS and incubated with Nile Red (Sigma) (diluted 1:1000 in sterile PBS 1X) to label lipid droplets for 30 min in the dark at room temperature. Next, coverslips were washed twice with sterile PBS and incubated overnight with a mouse monoclonal primary anti-*Toxoplasma gondii* antibody [SAG1/p30] (Abcam TP3 #ab8313, Cambridge, United Kingdom) (diluted 1:500 in PGN-0.01% saponin solution). Then, coverslips were washed thrice with sterile PBS and incubated with Alexa Fluor 488-conjugated anti-mouse IgG (Invitrogen, #A11001, Carlsbad, CA, USA) (diluted 1:500 in PGN-0.01% saponin solution) and TOPRO-3 Iodide (Life Technologies) (diluted 1:500 in PGN-0.01% saponin solution) for 1 h in the dark at room temperature to label tachyzoites of *T. gondii* and nuclei, respectively. Coverslips were mounted on glass slides and 20 images from different fields of each condition were analyzed by confocal fluorescence microscopy (Zeiss, LSM 510 Meta, Germany) with an inverted microscope (Zeiss Axiovert 200 M). Images were analyzed in Image J software version 1.50i (National Institutes of Health, USA, https://imagej.nih.gov/ij), and the data were expressed as the percentage of total intensity of LDs (% total intensity LDs/field) in each condition in comparison to untreated and uninfected cells (100% of total intensity LDs/field).

### Viability assay in human villous explants after COX-2 inhibitors treatment

The human villous explants were collected as described above and cultured for 24 h in medium with 10% FBS at 37 °C and 5% CO_2_. After 24 h, villous were tretated with meloxicam (10, 250, 500 and 1000 μg/mL) or celecoxib (5 and 10 μg/mL) in medium FBS-free for an additional 24 h. As control, villous explants were treated with only medium FBS-free (medium), corresponding to 100% viability. COX-2 inhibitors concentrations were based on a previous study by our research group^53^.

After 24 h of treatment, the supernatants were collected for the measurement of lactate dehydrogenase (LDH) enzyme levels as the expression of inhibitors toxicities, according to the manufacturer’s instructions (LDH Liquiform, Labtest Diagnostica S.A., Lagoa Santa, MG, Brazil). In this method, the enzyme LDH catalyzes the conversion of pyruvate to lactate in the presence of NADH. The absorbance was measured in a DU-70 spectrophotometer (Beckman, Brea, CA., USA) at 340 nm, for 2 min at 37 °C. The decrease in absorbance at 340 nm due to the oxidation of NADH is proportional to the activity of LDH in the sample. Data were expressed as U/L of enzyme activity. In addition, to verify explant integrity with COX-2 inhibitors, they were collected for morphological analysis using hematoxylin and eosin staining^56,62^. Two placentas were used, and two independent experiments with eight replicates were performed.

Furthermore, the MTT assay was performed on the villous^57,92,95^. After 24 h of treatment, the supernatants were collected and frozen at -80 °C for later measurements of cytokines. The villous were incubated with 20 µL of MTT (5 mg/mL) plus 180 µL medium with 10% FBS in the same culture condition at 37 °C and 5% CO_2_ for 4 h. The formazan crystals were solubilizied by adding a solution containing 10% SDS and HCl (0.01 M) at 37 °C and 5% CO_2_ for 18 h. The villous were removed from each well and the optical densities measured at 570 nm (Titertek Multiskan Plus, Flow Laboratories, McLean, VA, USA). Data were expressed as the percentage of viable villous (% viability of villous) in comparison untreated villous (100% of villous viability). Two placentas were used, and two independent experiments with eight replicates were performed.

### *T. gondii* intracellular proliferation in human villous explants after COX-2 inhibitors treatment

The villous explants were cultured in 96-well plates for 24 h in medium with 10% FBS at 37 °C and 5% CO_2_. Next, the villous were infected with *T. gondii* tachyzoites in a proportion of 1 × 10^6^ parasites to each well in medium FBS-free for 24 h. Next, the villous were treated with meloxicam (250 µg/mL) or celecoxib (5 µg/mL) in medium FBS-free for an additional 24 h, according to the results of the toxicity assay (LDH e MTT assay) and morphological analyses. As control, villous explants were infected and untreated (*T. gondii*). After 24 h, the supernatants were collected and frozen at -80 °C for later measurements of cytokines. In parallel, the *T. gondii* intracellular proliferation assay was performed in the infected cells by the β-galactosidase colorimetric reaction, as previously described^56,62^. The parasite intracellular proliferation in villous explants samples was performed by adding 150 µL RIPA buffer [50 mM Tris–HCl, 150 mM NaCl, 1% Triton X-100, 1% (w/v) sodium deoxycholate, and 0.1% (w/v) sodium dodecyl sulfate (SDS), pH 7.5] supplemented with protease inhibitor cocktail (Complete, Roche Diagnostic, Mannheim, Germany) to each villous and homogenizing the samples in ice for protein extraction. The homogenate was centrifuged at 21.000 × g for 15 min at 4 °C and the supernatant was collected to measure the protein total (µg/mL) using the Bradford assay^96^. Aliquots of 20 µL of each sample were used to determine *T. gondii* intracellular proliferation by β-galactosidase assay, as described above. Next, the data of number of tachyzoites were normalized according to the protein concentration of each villous, showing the number of tachyzoites per µg of tissue. The data were expressed as percentage (%) of *T. gondii* proliferation: the mean number of tachyzoites from controls (untreated and infected villous) corresponded to 100% of parasite proliferation, and the number of tachyzoites from each treatment condition was transformed into a percentage according to 100% of parasite proliferation from the control. Two independent experiments with eight replicates were performed.

### Cytokines and nitrite

The human cytokines IL-4, IL-6, IL-8, IL-10, TNF-α, MIF and TGF-β1 were measured in supernatants of BeWo and HTR-8/SVneo cells and villous explants by ELISA, according with manufacturer’s instructions (R&D Systems, Minneapolis, MN, USA; or BD Biosciences, San Diego, CA, USA). Data were expressed in pg/mL according to a standard curve of each cytokine for BeWo and HTR-8/SVneo cells, while for villous explants, the data were normalized according to the protein concentration of each villous. Then, for villous explants, the data about cytokines were obtained by the ratio between concentration of cytokines in pg/mL and concentration of total protein from Bradford assay in µg/mL, resulting in pg/mg of tissue^56,57,91^.

In addition, the cells supernatants were used to measure nitrite by the Griess method^97^. The supernatant were added to 96-well plates, incubated with Griess reagent (1% sulfanilamide dihydrochloride and 0.1% naphthylenediamide dihydrochloride in 2.5% H_3_PO_4_) for 10–20 min, and the absorbance was read in a plate reader (Titertek Multiskan Plus, Flow Laboratories, McLean, VA, USA) at 570 nm. The nitrite concentration was determined with reference to a standard curve of sodium nitrite (µM/mL).

### Statistical analysis

Statistical analysis was performed using the GraphPad Prism Sofware version 6.01 (GraphPad Software Inc., San Diego, CA, USA, https://www.graphpad.com). All data were expressed as mean ± standard error of mean (SEM). Differences between groups were assessed by One-Way ANOVA test with Sidak’s multiple comparison post-test for the parametric data, or Kruskall–Wallis test with Dunn’s multiple comparison post-test for the nonparametric data, or Student’s *t* test, when appropriate. Statistical differences were considered significant when *P* < 0.05.

### Ethical approval

The present research protocol using human tissue samples was performed in accordance with relevant guidelines and regulations, and the experimental protocols were approved by the Ethics Committee of the Federal University of Uberlandia, MG, Brazil, with approval number 1.585.342. A consent term was obtained from all subjects, and when the subjects were under 18, a parent and/or legal guardian was acquired. In addition, informed consent was obtained from all participants and/or their legal guardians.

## Supplementary Information

Supplementary Information 1.

Supplementary Information 2.

Supplementary Information 3.
