# Supplementary material for: Cyclooxygenase (COX)-2 modulates Toxoplasma gondii infection, immune response and lipid droplets formation in human trophoblast cells and villous explants
Source: Sci Rep. 2021 Jun 16;11:12709. doi: 10.1038/s41598-021-92120-3 (PMC8209052; doi:10.1038/s41598-021-92120-3)
Supplement: Supplementary file 1 — Supplementary Information 1. [file 41598_2021_92120_MOESM1_ESM.docx]

**Figure Supplementary S1 – Nitrite production in HTR-8/SVneo cells infected and treated with COX-2 inhibitors.** HTR-8/SVneo cells were infected or not with *T. gondii* for 3 h and treated or not with meloxicam or celecoxib for an additional 24 h. Next, the supernatants were collected for measurement of nitrite by Griess method (**A, B**). Data were shown as mean ± SEM from three independents experiments with eight replicates. Differences between groups were analyzed by One-Way ANOVA test with Sidak’s multiple comparison (**B**) or Kruskal-Wallis test and Dunn’s multiple comparison post-test (**A**) (GraphPad Prism Sofware version 6.01, https://www.graphpad.com). Significant differences in relation to untreated and uninfected cells (^*^uninfected medium), untreated and infected cells (^#^infected medium), and between concentrations of meloxicam (^$^), regardless of infection. Differences were considered significant when *P* < 0.05.

**Figure Supplementary S2 – LDs production in BeWo and HTR-8/SVneo cells infected with *T. gondii*.** BeWo (**A**) and HTR-8/SVneo (**B**) cells were infected or not with *T. gondii* FBS-free for 24 h. Next, cells were stained with Nile Red for visualization of lipid droplets (LDs) in confocal microscopy, and 20 images from differents fields for each condition were analyzed by the Image J software version 1.50i (National Institutes of Health, USA, https://imagej.nih.gov/ij). Data were presented as percentage (%) of total intensity of LDs/field in relation to uninfected cells (100% of total intensity LDs/field). Data were shown as mean ± SEM from two independents experiments with two replicates. Differences between groups were analyzed by Student’s *t* test (GraphPad Prism Sofware version 6.01, https://www.graphpad.com). Significant differences in relation to uninfected cells (^*^medium). Differences were considered significant when *P* < 0.05. Representative photomicrographs are shown in BeWo (**C**) and HTR-8/SVneo (**D**). Cell nucleus is labeled with TOPRO-3 (blue), lipid droplets are labeled with Nile Red (red) and *T. gondii* tachyzoites are labeled with Alexa Fluor 488-conjugated anti-mouse IgG (green). Scale bar: 20 µm
